# Efficient Gene Disruption via Base Editing Induced Stop in Newt *Pleurodeles waltl*

**DOI:** 10.3390/genes10110837

**Published:** 2019-10-23

**Authors:** Hao Cai, Zhelun Peng, Ruimin Ren, Heng Wang

**Affiliations:** College of Animal Sciences and Technology, Huazhong Agricultural University, Wuhan 430070, China; caihao@webmail.hzau.edu.cn (H.C.); pengzhelun@webmail.hzau.edu.cn (Z.P.); ruimin.ren@webmail.hzau.edu.cn (R.R.)

**Keywords:** base editing, sgRNAs, newt, genome

## Abstract

Loss-of-function approaches provide strong evidence for determining the role of particular genes. The prevalent CRISPR/Cas9 technique is widely used to disrupt target gene with uncontrolled non-homologous end joining after the double strand breaks, which results in mosaicism and multiple genotypes in the founders. In animal models with long generation time such as the salamanders, producing homozygous offspring mutants would be rather labor intensive and time consuming. Here we utilized the base editing technique to create the loss-of-function F0 mutants without the random indels. As a proof of principle, we successfully introduced premature stop codons into the *tyrosinase* locus and produced the albino phenotype in the newts (*Pleurodeles waltl*). We further demonstrated that the knockout efficiency could be greatly improved by using multiplex sgRNAs target the same gene. The F0 mutated animals showed fully loss-of-function by both genotyping and phenotyping analysis, which could enable direct functional analysis in the founders and avoid sophisticated breeding. This study not only presented the high efficiency of single base editing in a gigantic animal genome (>20 G), but also provided new tools for interrogating gene function in other salamander species.

## 1. Introduction

The salamanders comprise a group of more than 700 species of amphibians and constitute the order Caudata [1]. The order contains 10 families, among which are the true salamanders (family Salamandridae, including fire-belly newt, red-spotted newt, and Iberian ribbed newt) and mole salamanders (family Ambystomatidae, to which the axolotl belongs). They are historically important as biological research subjects in development, physiology, behavior, and evolution. However, the salamander’s vast genomes (ranging from 14 and 120 Gb) and the lack of genetic interrogation tools hindered their experimental applications [2]. The salamander research finally gained momentum in recent years with the newly assembled genomes of axolotl [3] and Iberian ribbed newt [4], as well as the ever-advancing gene and cell manipulation techniques [5]. Nevertheless, the gold standard of gene analysis with loss- and gain-of-function procedures are inconvenient and still need to improve in salamanders.

The prevalent gene editing tool, clustered regularly interspaced short palindromic repeats-associated nucleases (CRISPR)/Cas, was widely used to disrupt gene function. It creates DNA double-strand breaks (DSBs) followed by non-homologous end joining (NHEJ) to generate random indels which leads to functional loss of the gene. Previous studies have applied CRISPR/Cas knockout to the axolotls and newts and yielded excellent insights into the underlying mechanisms responsible for the unique features of salamanders [6,7,8], particularly related to the evolutionary developmental biology. The recent base editing is the new technique based on CRISPR/Cas9, enables the single base pair conversion in specific locus of the genome, without causing any DSBs thus limiting the DNA damage [9]. The base editor, usually composed of the cytidine/adenine deaminase and catalytically defective Cas9 with the sgRNA, were successfully used to introduce single-nucleotide polymorphisms of interest in different organisms and manipulate the disease-causing mutations in human cells [10]. Whether the specific and accurate single base pair conversion could also be achieved in the extraordinary genomes of salamanders remains unknown.

In this study, we tested the possibility of base editing in salamanders by using the induction of stop codons (iSTOP) knockout strategy in the Iberian ribbed newt (*Pleurodeles waltl*). The gene inactivation is realized by precisely converting the four codons (CAA, CAG, CGA, or TGG) into stop codons (TAG, TAA, or TGA), by the cytidine deaminase (C-to-T) in the base editor complex, and subsequently the early termination of the translation. We initially focused on the single copy newt *tyrosinase* (tyr) gene as its protein product is the rate-limiting enzyme in the synthesis of melanin [11]. The loss-of-function mutations in *tyr* locus could produce albino animals with no pigmentation and develop normally, which is a convenient phenotypic readout of successful gene disruption. We designed sgRNAs to target the first exon of *tyr* gene and utilized both the canonical CRISPR/Cas9 (indel mutation) and the latest base editing means (iSTOP) to achieve loss-of-function. Our results showed that the base editing technique and multiplex sgRNA strategy could efficiently induce iSTOP to inactivate the *tyr* gene to create albinism phenotype. The base editing is a potent way to safely and stably generate gene modified salamanders. 

## 2. Materials and Methods 

### 2.1. Synthesis of Base Editor mRNA and sgRNAs

The fourth generation of base editor was used in this study. pCMV-AncBE4max (AncBE4max) was a gift from Dr. David Liu (Addgene plasmid # 112094). The base editing window of AncBE4max was around positions four to eight of the protospacer, counting the PAM site as positions 21–23. The plasmid was linearized by using restriction enzyme BbsI and purified with ethanol precipitation. In vitro transcription (IVT) was performed with mMESSAGE mMACHINE T7 ULTRA kit (ThermoFisher Scientific, AM1345, Waltham, MA, USA). The transcribed mRNA was purified by lithium chloride precipitation and the concentration was adjusted with RNase-free water. All the sgRNAs are targeting *tyr* exon 1 to maximize the early translation termination. The sgRNA#1, #2, and #5 were designed to convert codon CAG into TAG within the editing window. The sgRNA#3 and #4 were designed to convert ACC in the anti-sense strand into ATC/ACT/ATT and thus TAG/TGA/TAA in the sense strand. The sgRNA sequences were: sgRNA#1: GTGGCCAGCTCTCTGGCCG; sgRNA#2: GACCCCAGTTTCCGTTTTC; sgRNA#3: GGTGCCACGGCAGGAAGGC; sgRNA#4: CCTCCAGTCCCAGTACGGGA; and sgRNA#5: CGACCAGCTGATGGGGGAC. The GeneArt™ Precision gRNA Synthesis Kit (ThermoFisher Scientific, A29377, Waltham, MA, USA) was employed to synthesize the sgRNAs. The IVT was performed according to the manufacturer’s instructions. The sgRNAs concentration was adjusted to 50–100 ng/μL and sgRNAs was kept at −80 °C until use. 

### 2.2. Animal Care and Egg Injections

Iberian ribbed newts (*Pleurodeles waltl*) were kept in tap-water tanks at 22–24 °C under natural light cycles. The larvae were fed with hatched artemia every day and the juvenile/adult newts were fed with frozen blood worms and pellets every other day. Natural breeding was promoted and the egg laying was induced by human chorionic gonadotropin injection of females [12]. The one cell stage eggs were collected and manually de-jellied before injection. All procedures were carried out in accordance with the Institutional Animal Care and Use Committee of Huazhong Agricultural University (ethics approval number: 2018-0125).

The amount of AncBE4max/spCas9 mRNA and sgRNAs for each egg were optimized according to previous published protocols [4]. The amount of 250 pg AncBE4max/spCas9 mRNA and 50 pg sgRNAs per egg were used. For the multiplex sgRNAs injections, 16.7 pg (low concentration) or 50 pg (high concentration) of each sgRNAs were mixed together. Then AncBE4max mRNA were added and adjusted to the appropriate volume for injection. Using a PV830 Pneumatic PicoPump (World Precision Instruments, Sarasota, FL, USA), the mixed solution was injected into the animal hemisphere of fertilized eggs, in 6% Ficoll with 1×Holtfreter’s solution. The injected embryos were maintained in 0.1×Holtfreter’s solution at 24 °C. 

### 2.3. Animal Genotyping and Phenotyping

The stage 36 developing larvae [13] were anesthetized with 0.01% tricaine and the genomic DNAs were extracted with TIANamp Genomic DNA Kit (Tiangen, DP304, Beijing, China). PCRs were performed with Premix Taq™ DNA Polymerase (Takara, RR003A, Kusatsu, Japan) and the PCR amplicons covering the base editing region were either sequenced directly by Sanger sequencing or subcloned into TA cloning vector pMD19T (Takara, Kusatsu, Japan) for further analysis. The PCR conditions were: 95 °C for 1 min, 35 cycles of 95 °C for 15 s, 60 °C for 10 s, 72 °C for 60 s, and a final step at 72 °C for 5 min. The genotyping primers are: Forward: TGGGGTGTTGCAGGTTAGCT and reverse: TGGCCTTTGCATGTTGGGAC. The animal pictures were taken with the Olympus SZX16 stereomicroscope (Olympus, Tokyo, Japan). 

## 3. Results

### 3.1. Strategy for Designing Premature Stop Codon for Target Gene Knockout in Newt

**Figure 1:**
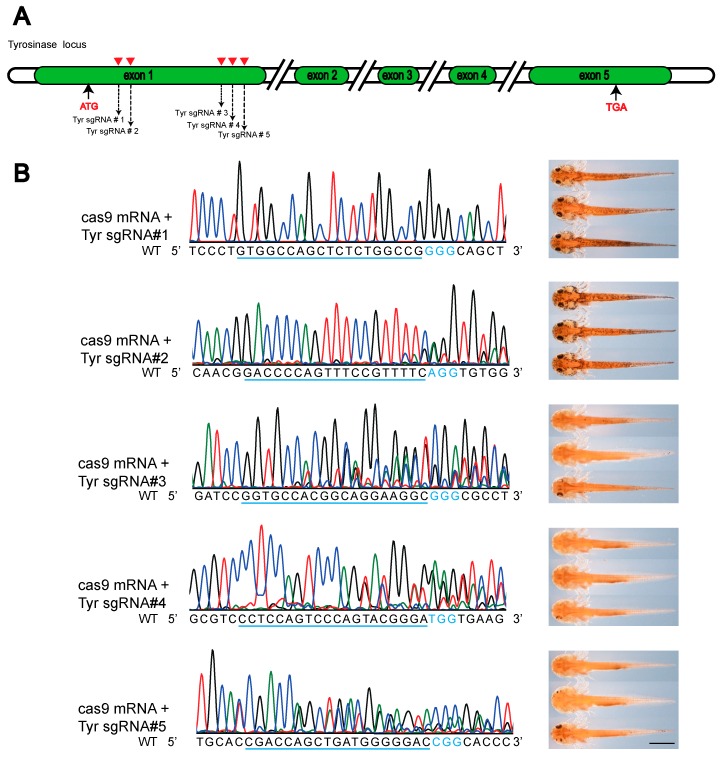
The scheme of the clustered regularly interspaced short palindromic repeats-associated nucleases (CRISPR)/Cas9 and base editing mediated *tyrosinase* (*tyr*) knockout in newt. (**A**) Five sgRNAs were designed close to the ATG start codon of the *tyr* exon 1; (**B**) The genotype and phenotype of the *tyr* knockout animals produced with the canonical CRISPR/Cas9 technique. The sanger sequencing chromatograms (left panels) and the representative pictures of corresponding animals (right panels) were shown. The predicted protospacer sites were underlined. The NGG pam site was highlighted in blue. The overlapping peaks starting around the PAM site indicated multiple mutant alleles were created. Scale bar: 2 mm. sgRNA (small guide RNA).

The detailed scheme of the iSTOP design was shown in Figure 1. According to the principle of iSTOP, we design five sgRNAs to target the first exon of the newt tyrosinase (*tyr*) gene (Figure 1A). We first tested if these sgRNAs could specifically and effectively target the *tyr* locus by microinjecting the SpCas9 mRNA and sgRNAs into fertilized newt eggs (single-stage embryos). The CRISPR/Cas9 system can continuously operate at the target during embryonic development, leading to mosaicism of the introduced mutations. If the CRISPR/Cas9 destroy the *tyr* gene, the pigmentation phenotype can be visually identified. Thirteen days later, we observed albino animals in three out of five groups of sgRNA injected embryos, indicating that only the sgRNA#3, #4, and #5 are operative. The knockout efficiency varies in different groups (sgRNA#1: 0/17, sgRNA#2: 0/15, sgRNA#3: 16/24, sgRNA#4: 39/50, and sgRNA#5: 11/20). Then we extracted DNA from individual animals from each group and PCR amplified the target region. Indeed, the sanger sequencing chromatograms showed complicated mosaic mutations adjacent to the PAM site, indicate multiple indels occurs in the same animal (Figure 1B). According the PCR sequencing results, four out of five (sgRNA#2, #3, #4, and #5) groups have successfully created frame shift mutations, but we only found obvious albinism in sgRNA#3, #4, and #5 groups, indicating that sgRNA#2 was inefficient. Nevertheless, we continued to test all five sgRNAs in the base editing experiments. 

### 3.2. Generation of Albino Newt with Base Editing-induced Stop Codon in Tyrosinase Gene

**Figure 2:**
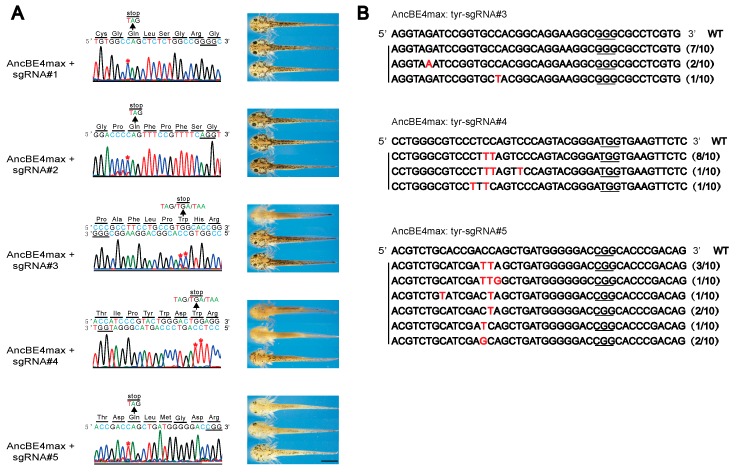
The base editing mediated induction of stop codons (iSTOP) in the *tyr* gene produces albino animals: (**A**) The genotyping and phenotyping results of the base edited animals. The sequencing chromatograms and the animal pictures showing the base pair conversions created new stop codons (left panels) and varies degrees of albinism (right panels). The asterisks (*) indicate the C-to-T conversions. Scale bar: 2 mm; (**B**) The alignment of the genomic sequence in the base editing region. Ten TA clones were sequenced from one base edited individual and the frequency of each genotype was shown in the parentheses. The base pair conversions are showing in red. The PAM sites were underlined.

To explore whether the base editing tool is working in newt, we performed similar procedures by co-injecting AncBE4 mRNA and sgRNAs into fertilized eggs. After PCR sequencing, we identified C-to-T conversions in the specific loci as expected in four out of five (sgRNA#2, #3, #4, and #5) groups (Figure 2A). However, the sgRNA#2 injected embryos only showed modest base pair conversion and thus not exhibit apparent albinism (Figure 2A). The results indicated that the AncBE4max is functional and the iSTOP strategy is feasible in the newt. The detailed sequence analysis with the cloning of PCR products showed that only base pair conversions occurred around the editing window. No deletions or insertions were observed in the target region (Figure 2B). 

Next, we examined the pigmentation in the eyes as the reference to determine the knockout efficiency in the base edited animals. A total of 10%, 66%, and 13% of animals injected with sgRNA#3, #4, and #5 showed missing melanin in at least one eye (Table 1). In comparison, the CRISPR/Cas9 with the most efficient gRNA#4 yielded 78% (39/50) of animals showing loss of pigment in one or two eyes, indicate that the efficiency of base editing mediated knockout is comparable to the indel derived knockout by canonical CRISPR/Cas9.

### 3.3. Multiplex sgRNAs Improves the Knockout Efficiency

**Figure 3:**
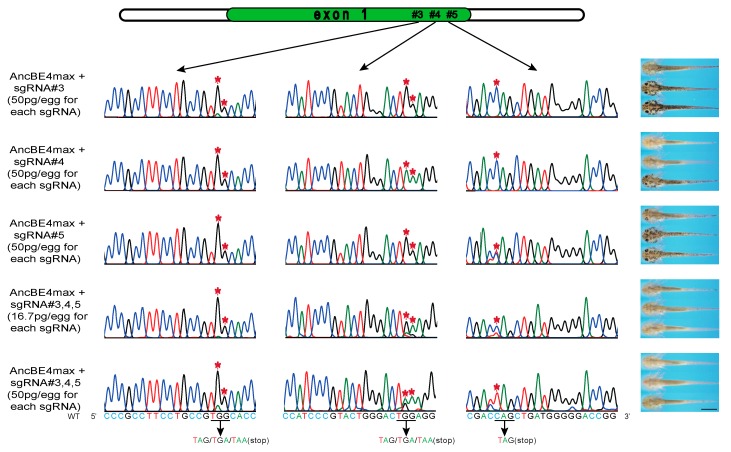
Multiplex sgRNAs could improve the *tyr* knockout efficiency. The single sgRNAs or multiplex sgRNAs were co-injected with the base editor mRNA into the fertilized eggs and the genotyping and phenotyping were performed 13 days later. The PCR sequencing chromatogram of the three editing sites from the same animal were shown in the left panels. The representative pictures of animals were shown in the right panels. The base pair conversions could be detected in all three editing sites of the individuals with the multiplex sgRNAs injected. Asterisks (*) indicate the base pair conversions. Scale bar: 2 mm.

After verifying the actions of sgRNAs and AncBE4max on iSTOP, we continued to test whether the multiple sgRNAs strategy is possible to improve the efficiency of gene deletion. The selected sgRNA#3, #4, and #5 were injected into the one-cell embryos separately or combined while the AncBE4max mRNA amount remain constant. At 13-day post injection, we found that the single sgRNAs can induce at most half of animals to completely lose the pigmentation, but multiple sgRNAs together can produce 70% of the animals with fully loss of pigment. Co-injection of multiple-sgRNAs with “high concentration” has similar gene disruption effect compare to “low concentration”, these results indicate that the improving of knockout efficiency is not due to the increase of the substance of sgRNAs, but to multiple-sgRNAs (multiple stops) strategy (Table 1). The DNA genotyping analysis also confirmed that the base pair conversion occurs at all three base editing loci in each injected animal (Figure 3), thus increased the likelihood of premature translation stop. These data further suggest multiple sgRNAs facilitated iSTOP conversion and gene disruption, which allows direct phenotype analysis of the founder animals.

## 4. Discussion

As detailed above, the base editing works efficiently in newts by inducing targeted C-to-T conversion that can introduce a premature stop codon into a protein-coding sequence, providing a means of generating loss-of-function mutants. Current method presents several advantages compared to the canonical CRISPR/Cas9 mediated gene disruption technology. First, base editing does not elicit any DSBs, whose formation is particularly deleterious in the early stage of animal development [14]. Furthermore, base editing can inactivate the coding gene by introducing as little as only one-point mutation to maximally keep the gene intact, which will minimally perturb the epigenetic regulation around the gene. This is probably crucial in salamanders because of the enrichment of non-coding elements, including miRNAs [4] and transposable elements [3,15], in the gigantic genomes. Finally, the current multiplex iSTOP strategy can greatly increase the production of isogenic founder animals for direct functional analysis. It is particularly favorable for the salamander research because of the long generation time and complicated mating could be avoided. 

Latest studies showed that cytosine but not adenine base editors have undesired off-target effects on DNA in mice [16] and rice [17] and even RNA in human cells [18]. It raised serious concerns for the application of the cytosine base editor, especially for its clinical translation. In this study, we did not perform the off-target analysis because of the inadequate genomic resources. The preliminary TA cloning sequencing results showed that additional types of base pair conversions (A-to-G, C-to-G) other than C-to-T occurred close to the editing region of sgRNA#5, although the incidence rates were low (3/16). Nevertheless, the *tyr*-base edited animals developed and metamorphosed normally compare to the wild type ones (Figure S1), indicating very limited cytotoxicity [19]. Despite these important observations, it remains to be determined whether cytosine base editing cause unwanted mutations in newts through in-depth analysis.

To our knowledge, this report presents the evidence showing the largest animal genome ever been successfully base edited. With the constant engineering to the base editors to improve the editing window, efficiency, and accuracy, the next generations of base editing technique will undoubtedly be safer and easier to use [20]. The current multiplex sgRNA iSTOP strategy can be easily adapted to other loss-of-function assays and the *tyr* base editing could serve as the technical control. 

## 5. Conclusions

Altogether, our work established the base editing mediated iSTOP as a robust and efficient gene disruption technology in newt and provided invaluable tools to analyze the interesting genes identified from the growing omics data of different salamander studies.

## Figures and Tables

**Table 1:** Phenotypic penetrance of base edited animals with single sgRNA (small guide RNA) or multiplex sgRNAs.

sgRNAs ^1^	Injected Eggs	Survived Embryos	Phenotype ^2^		
			Strong	Weak	None
sgRNA#3 (50 pg)	115	82	7 (8.5%)	1 (1.2%)	74 (90.2%)
sgRNA#4 (50 pg)	143	62	31 (50%)	10 (16.1%)	21 (33.9%)
sgRNA#5 (50 pg)	128	32	2 (6.3%)	2 (6.3%)	28 (87.5%)
sgRNA#3 #4 #5 (16.7 pg each)	106	40	28 (70%)	8 (20%)	4 (10%)
sgRNA#3 #4 #5 (50 pg each)	128	81	53 (65.4%)	15 (18.5%)	13 (16.1%)
Control (ancBE4max only)	108	92	0	0	92 (100%)

Note: ^1^ The amount of ancBE4max mRNA was consistently at 250 pg/egg. The amount of sgRNAs was 50 pg/egg for single sgRNA injections and 16.7 pg/egg or 50 pg/egg for each sgRNA in combined injections. ^2^ The albino phenotype (stage 36) was categorized as “strong” (both eyes missing pigmentation), “weak” (one eye missing pigmentation), “none” (both eyes are black). The proportion of each phenotype are given in brackets.

## Supplementary Materials

The following are available online at https://www.mdpi.com/2073-4425/10/11/837/s1, Figure S1: Base edited newts developed normally compare to the wild types.
