# Supplementary material for: Efficient Gene Disruption via Base Editing Induced Stop in Newt Pleurodeles waltl
Source: Genes (Basel). 2019 Oct 23;10(11):837. doi: 10.3390/genes10110837 (PMC6895984; doi:10.3390/genes10110837)
Supplement: Supplementary file 1 [file genes-10-00837-s001.pdf]

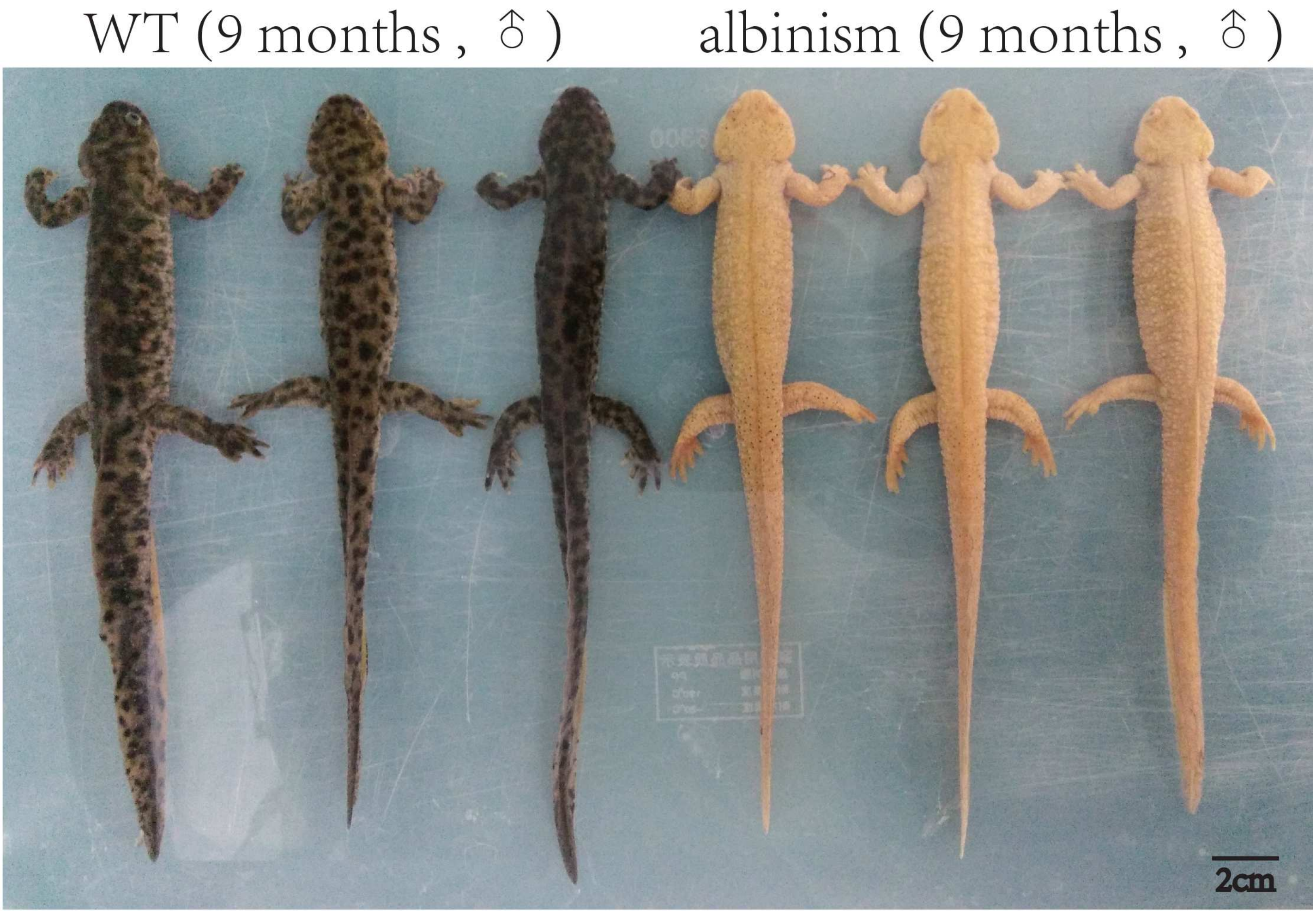

Figure S1. The base edited newts developed normally compare to the wild type animals. The multiplex sgRNAs induced iSTOP knockout in *tyrosinase* gene produced albino animals.
